# Long‐term headache after spontaneous intracerebral haemorrhage

**DOI:** 10.1111/ene.16247

**Published:** 2024-02-13

**Authors:** Ugur Gurol, Giuseppe Scopelliti, Charlotte Cordonnier, Laurent Puy

**Affiliations:** ^1^ Inserm, CHU Lille, U1172 ‐ LilNCog ‐ Lille Neuroscience and Cognition University of Lille Lille France; ^2^ Neuroscience Institute The University of Chicago Chicago Illinois USA

**Keywords:** headache, intracerebral haemorrhage, stroke

## Abstract

**Background:**

Though headache is commonly observed after stroke and may affect survivors' quality of life, it has rarely been studied after spontaneous intracerebral haemorrhage (ICH). In a cohort of ICH survivors, we assessed the long‐term prevalence and determinants of headache.

**Methods:**

We screened consecutive ICH survivors enrolled in the prospective, single‐centre Prognosis of Intracerebral Haemorrhage study for headache 1, 3, and 6 years after ICH, according to the International Headache Society's criteria. Depressive and anxiety symptoms severity was measured at 1‐year follow‐up. Variables associated with the presence of headache 1 year after ICH were analyzed using univariate and multivariable models.

**Results:**

Among the 146 patients included in this study, 31 (21%), 25 (19%), and 14 (20%) patients reported headache at 1‐, 3‐, and 6‐year follow‐up, respectively. In an age‐adjusted model, patients with headache at ICH onset (adjusted odds ratio [aOR] 2.75; 95% CI 1.02–7.42) and previous history of headache (aOR 4.60; 95% CI 1.74–12.1) were associated with headache at 1‐year follow‐up. Patients with headache were more likely to report depressive and anxiety symptoms at 1‐year follow‐up (both *p* < 0.02).

**Conclusions:**

One in five ICH survivors suffered from headache and patients who reported headache at ICH onset were especially at risk.

## INTRODUCTION

Headache has been characterized as a manifestation of post‐stroke pain, with prior studies reporting prevalence varying from 7% to 23% – at different follow‐up times – within the first 3 years after index stroke [1, 2, 3, 4, 5]. Of note, most of these studies were biased towards ischaemic stroke survivors and characterized headache in the broader context of post‐stroke pain. In particular, the prevalence and associated factors of headache after spontaneous intracerebral haemorrhage (ICH) remain poorly investigated. In a cohort of 1‐year spontaneous ICH survivors, we sought to evaluate the long‐term prevalence of post‐ICH headache. We also investigated demographic, clinical, and radiological determinants of post‐ICH headache.

## METHODS

### Participants and study design

We included consecutive patients with spontaneous ICH enrolled in the prospective, observational, single‐centre ‘Prognosis of InTraCerebral Haemorrhage’ (PITCH) study from 2004 to 2009 who were able to complete our headache questionnaire 1 year after index ICH. Patients that had not been screened at 1 year but were assessed at 3 years and/or 6 years were also included at these respective time points. We recorded the presence of headache at ICH onset and any previous history of headache, along with other anamnestic, clinical, and radiological baseline factors. Patient enrollment criteria, as well as the nature of the clinical and radiological evaluations performed, have been detailed in previous studies [6].

### Follow‐up evaluation

Each patient was invited to be followed up at prespecified study time points following the index ICH, as previously described [6]. We evaluated patients for headache at 1‐, 3‐, and 6‐year follow‐up using an in‐house, neurologist‐administered headache questionnaire that surveyed the presence of headache before, during, and after ICH. We used the criteria of the *2nd Edition of the International Classification of Headache Disorders* to diagnose the presence and type of headache [7]. The following two features were also recorded at 1‐year follow‐up: (i) depressive symptoms severity, employing the observer‐rated Montgomery‐Åsberg Depression Rating Scale (MADRS) and (ii) anxiety symptoms severity, using the self‐administered Anxiety subscale of the Hospital Anxiety and Depression Scale (HADS‐A) [8, 9].

### Statistical analyses

Clinical and radiological characteristics of 1‐year survivors included in and excluded from the study were compared using the chi‐square test (or Fisher's exact test when appropriate) for categorical measures and the Mann–Whitney *U* test for continuous measures to assess whether included patients were representative of the entire ICH survivors' cohort. Prevalence of headache was calculated 1, 3, and 6 years after index ICH. We investigated univariate associations between headache 1 year after ICH and clinical and radiological features using the same testing scheme as for the included versus excluded analyses. We performed one multivariable logistic regression model to analyze factors associated with headache at 1‐year follow‐up. The independent variables selected for the model were pre‐ICH conditions and baseline measures associated with a *p*‐value < 0.10 in univariate analyses. Collinearity was evaluated by computing variance inflation factor (VIF) scores, with the alert threshold VIF ≥ 2.5. We conducted all statistical tests at the two‐tailed *α* level of 0.05 using R version 4.1.2 (https://www.r‐project.org).

## RESULTS

At one‐year follow‐up, 255 (45.5%) of the original 560 patients with spontaneous ICH enrolled in the PITCH cohort were alive. Of the survivors, 109 patients (42.7%) were unable to undergo headache screening; they were older than included patients (*p* < 0.05; Table S1: Data S1). In total, 146 patients were included in this study.

**FIGURE 1:**
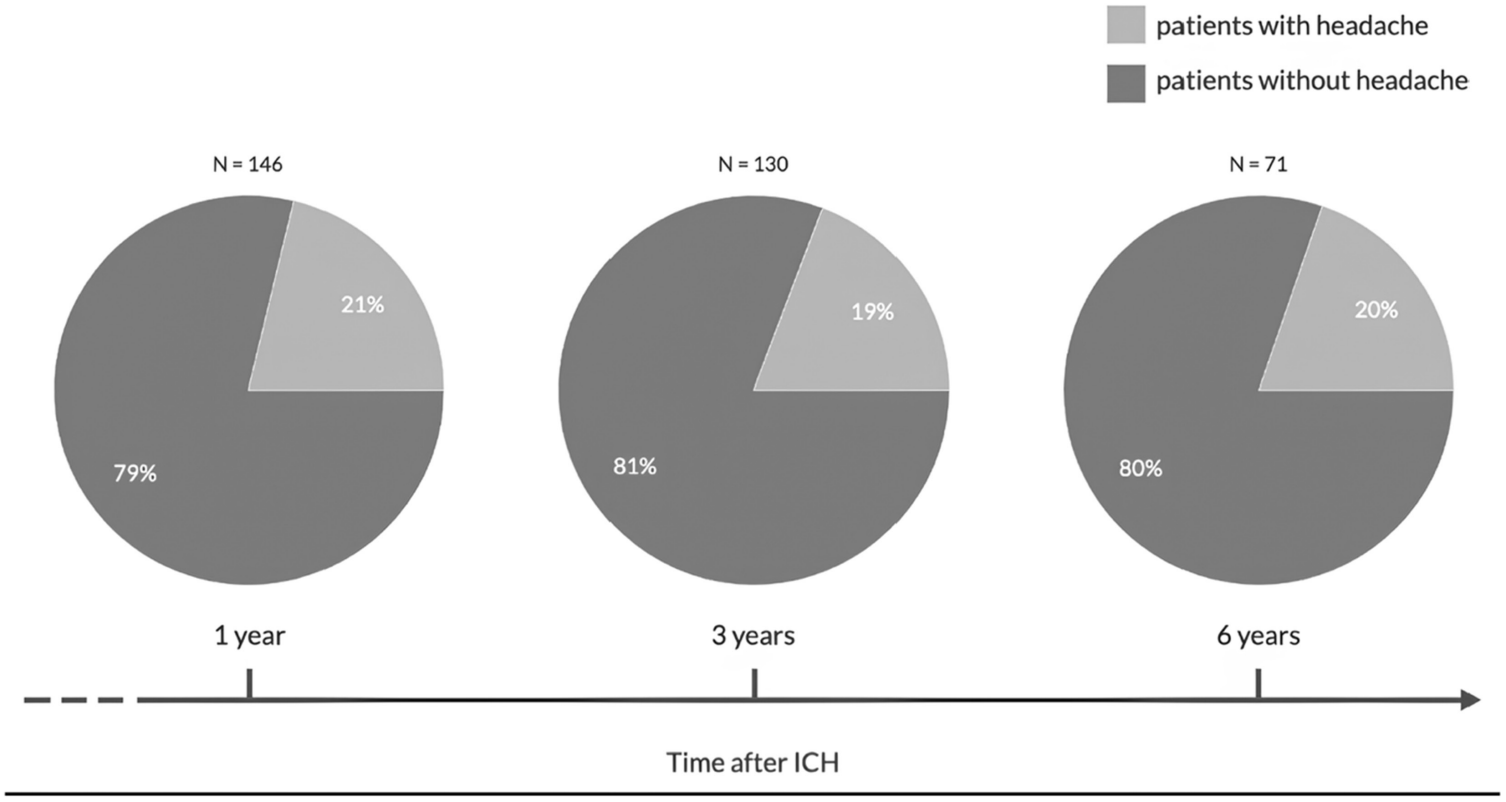
Prevalence of headache at 1, 3, and 6 years after index intracerebral haemorrhage (ICH).

At the 3‐ and 6‐year follow‐up, respectively, 130 and 71 patients were alive and screened for headache (Figure 1). Thirty‐one (21.2%; 95% confidence interval [CI] 14.6–27.8), 25 (19.2%; 95% CI 12.4–26.0), and 14 (19.7%; 95% CI 10.4–29.0) patients had headache at 1‐, 3‐, and 6‐year follow‐up, respectively (Figure 1).

At 1‐year follow‐up of the 102 patients without previous history of headache, 12 (12%) had developed new‐onset headache. Meanwhile, among the 44 patients with previous history of headache, 19 (43%) complained about headache at 1‐year follow‐up, 5 of whom (26%) reported that symptoms had worsened after the index ICH.

Table 1 displays univariate associations between headache at 1‐year follow‐up and demographic, clinical, and radiological characteristics. Younger age (*p* = 0.047), previous history of headache (*p* < 0.001), and headache at ICH onset (*p* = 0.006) were associated with presence of headache at 1‐year follow‐up. Female sex was not associated with presence of headache.

**TABLE 1:** Patient characteristics and headache at 1‐year follow‐up.

Baseline clinical and radiological features	Total (*n* = 146)	Without headache (*n* = 115)	With headache (*n* = 31)	*P*‐value
Age, median (IQR)	64.0 (53.2–75.7)	66.3 (53.6–76.9)	56.0 (51.7–72.1)	0.047
Male sex, *n* (%)	87 (59.6)	68 (59.1)	19 (61.3)	0.991
Hypertension, *n* (%)	92 (63.0)	73 (63.5)	19 (61.3)	0.989
Previous history of headache, *n* (%)	44 (30.1)	25 (21.7)	19 (61.3)	<0.001
Migraine, *n* (%)	18 (12.3)	11 (9.6)	7 (22.6)	0.065*
Tension headache, *n* (%)	14 (9.6)	8 (7.0)	6 (19.4)	0.077*
Unspecified, *n* (%)	12 (8.2)	6 (5.2)	6 (19.4)	0.021*
Headache at ICH onset, *n* (%)	32 (24.8)	20 (19.2)	12 (48.0)	0.006
ICH volume at admission,^a^ median (IQR)	7.3 (1.7–20.0)	5.7 (1.8–17.8)	13.9 (2.2–21.6)	0.222
ICH cortical involvement, *n* (%)	46 (31.5)	33 (28.7)	13 (41.9)	0.234
Lobar ICH location, *n* (%)	51 (34.9)	40 (34.8)	11 (35.5)	1
ICH subarachnoid extension, *n* (%)	14 (9.6)	12 (10.4)	2 (6.5)	0.735*
1‐Year follow‐up affective symptoms				
MADRS score, median (IQR)	4 (0–10)	3 (0–8)	10 (3–16.5)	0.006
HADS‐A score, median (IQR)	3 (0–5)	2 (0–4.5)	4 (1.5–8.5)	0.014

Abbreviations: HADS‐A, Hospital Anxiety and Depression Scale (Anxiety subscale); ICH, intracerebral haemorrhage; IQR, interquartile range; MADRS, Montgomery‐Åsberg Depression Rating Scale.

^a^ Expressed in millilitres (mL).

* Fisher's exact test performed.

In a multivariable logistic regression model, both headache at ICH onset (adjusted odds ratio [aOR] 2.75; 95% CI 1.02–7.42) and previous history of headache (aOR 4.60; 95% CI 1.74–12.1) were significantly associated with headache at 1‐year follow‐up. Interestingly, none of the ICH characteristics were associated with the presence of headache. Patients with headache at 1‐year follow‐up had more severe depressive (MADRS score [IQR] = 10 [3–16.5] vs. 3 [0–8], *p* = 0.006) and anxiety symptoms (HADS‐A score [IQR] = 4 [1.5–8.5] vs. 2 [0–4.5], *p* = 0.014) compared to patients without.

## DISCUSSION

One in five ICH survivors reported headache throughout the 6 years of post‐ICH follow‐up. Headache at onset was a key determinant of long‐term post‐ICH headache and should be considered to identify patients at risk. Symptoms of anxiety and depression frequently coexisted with headache at 1‐year follow‐up.

In this study, we investigated a condition that has rarely been assessed in ICH. During the entire follow‐up period, post‐ICH headache was observed in 20% of survivors. This result was consistent with the 7%–23% post‐stroke headache prevalence documented in the literature [2]. The only other prior investigation of post‐ICH headache differed from ours in many of these respects as it was a retrospective, small‐centre study that included cases of non‐spontaneous ICH [1]. Of note, a recent review of headache in general, non‐clinical populations estimated the global prevalence of headache to be 52% [10]. However, it is difficult to compare the prevalence of headache across different studies due to variations in headache definition, study design and patient inclusion criteria among other factors.

Unsurprisingly, patients were more likely to report headache 1‐year after ICH if they already had a history of headache before the index event. One in four of these patients with previous history of headache reported worsening symptoms at 1‐year follow‐up, suggesting a further deterioration of headache post‐ICH. Contrary to previous literature on headache in the general population and in various conditions, we did not find an association between female sex and headache at 1‐year follow‐up [1, 3].

Notably, we found that the complaint of headache as a symptom of ICH onset was significantly associated with 1‐year post‐ICH headache in a multivariable model adjusted for age and previous history of headache. This association was also described in a previous work on ischaemic stroke [4]. Our results underscore the importance of screening for headache at ICH onset in order to proactively identify and manage post‐ICH headache.

ICH survivors with headache were more likely to have symptoms of anxiety and depression compared with patients without. The associations between headache, especially migraine, and affective disorders have been well‐documented [11]. Furthermore, mood disorders, especially depression, have previously been identified as significant determinants of quality of life in stroke survivors [12]. Thus, screening for and treating mood disorders may simultaneously help to alleviate headache symptoms in ICH survivors and improve their quality of life.

Little is known about the pathophysiological processes underlying post‐stroke headache; however, prior studies have speculated on headache occurring at stroke onset. While some have hypothesized that headache at stroke onset is related to the effect of the mechanical stretch caused by the haematoma on pain‐sensitive intracranial structures, the lack of an association between ICH volume and headache at 1‐year follow‐up in our cohort would suggest that this mechanism is unlikely to be responsible for long‐term headache [13, 14]. In fact, headache at 1‐year follow‐up was not associated with any of the radiological characteristics we tested, including bleeding topography and subarachnoid extension.

Going forward, the priority for clinicians regarding the management of headache after ICH should be developing a more thorough characterization of patients' headache after ICH and exploring the pathophysiological link between acute ICH and long‐term headache. In this regard, prospective studies of larger cohort size and analyses focused on new‐onset headache after ICH will be particularly valuable [15].

The main strength of this study is the extensive, long‐term clinical and radiological characterization of the included patients. This study also has certain limitations. As this is a single‐centre study, its external validity may be challenged. However, a previous comparison of the PITCH cohort with a population‐based ICH registry showed that recruiting bias was unlikely to have affected our sample [7]. We further recognize that the exclusion of 43% of 1‐year ICH survivors who were unable to undergo the headache questionnaire may have hindered the generalizability of our results. Even though included patients were younger than those excluded, our data suggest that these more mildly affected patients, in whom headache is a more frequent complaint, are more likely to benefit from being screened.

## CONCLUSIONS

One in five ICH survivors suffered from headache in the following years. Acute headache at ICH onset was a key anamnestic feature to identify these patients. In light of the associations between headache and depressive and anxiety symptoms, specific management of headache could improve the quality of life of ICH survivors.

## ETHICS STATEMENT

The study protocol was considered observational by the Internal Review Board of the Lille University Hospital that gave ethics approval for this study. Patients (or their relatives or primary caregiver) gave informed consent for the study.

## AUTHOR CONTRIBUTIONS

**Laurent Puy:** Conceptualization; investigation; writing – original draft; methodology; supervision. **Ugur Gurol:** Conceptualization; writing – original draft; methodology; formal analysis; data curation; investigation. **Giuseppe Scopelliti:** Investigation; writing – review and editing; methodology. **Charlotte Cordonnier:** Writing – review and editing; methodology; supervision.

## CONFLICT OF INTEREST STATEMENT

U.G.: none. G.S.: none. L.P. declares the following interests: speaker fees from Novo Nordisk. C.C. declares the following interests: speaker fees from Amgen and Bristol‐Myers Squibb; international RCT Steering committees for Biogen and Bayer; grant funding from the French Ministry of Health (Programme Hospitalier de Recherche Clinique): A3ICH and TICH‐3 Fr studies.

## Supporting information


Data S1.

## Data Availability

The data that support the findings of this study are available from the corresponding author upon reasonable request.
