# Supplementary material for: Long‐term headache after spontaneous intracerebral haemorrhage
Source: Eur J Neurol. 2024 Feb 13;31(5):e16247. doi: 10.1111/ene.16247 (PMC11235855; doi:10.1111/ene.16247)
Supplement: Supplementary file 1 — Data S1. [file ENE-31-e16247-s001.docx]

**Supplementary materials**

**Supplementary Methods:**

***Baseline demographic and clinical evaluation***

We collected data on demographic traits, vascular risk factors, and history of stroke (ischemic or hemorrhagic) using previously specified definitions.^1,2^

***Computed tomography imaging***

All patients enrolled in the study underwent a computed tomography (CT) scan at admission to determine ICH location and volume. We classified ICH location as either lobar (frontal, temporal, parietal, occipital lobes) or non-lobar (lenticular or caudate nucleus, thalamus, internal or external capsule, brainstem, cerebellum). Subarachnoid extension and cortical involvement of ICH were evaluated depending on whether or not the hematoma extended into these anatomical regions. We calculated ICH volume in accordance with the ABC/2 method.^3^

| **Characteristics of interest** | **Total**  **(n = 255)** | **Included**  **(n = 146)** | **Excluded**  **(n = 109)** | **p-value** |
| --- | --- | --- | --- | --- |
| Age, median (IQR) | 67.6 (55.0-77.1) | 63.6 (53.2-76.0) | 69.7 (60.3-78.6) | 0.005 |
| Male sex, n (%) | 146 (57.3) | 87 (59.6) | 59 (54.1) | 0.457 |
| Hypertension, n (%) | 166 (65.1) | 92 (63.0) | 74 (67.9) | 0.499 |
| Previous IS or ICH, n (%) | 35 (13.7) | 16 (11.0) | 19 (17.4) | 0.193 |
| Pre-ICH mRS score ≥3, n (%) | 41 (16.1) | 21 (14.4) | 20 (18.3) | 0.496 |
| Baseline NIHSS score, median (IQR) | 8.0 (4.0-15.0) | 8.0 (3.0-15.0) | 8.0 (4.0-16.0) | 0.324 |
| ICH volume,^a^ median (IQR) | 7.2 (2.2-20.0) | 7.1 (1.8-19.8) | 7.2 (2.8-21.5) | 0.410 |
| ICH cortical involvement, n (%) | 89 (34.9) | 46 (31.5) | 43 (39.4) | 0.237 |
| Lobar ICH location, n (%) | 97 (38.0) | 51 (34.9) | 46 (42.2) | 0.292 |
| ICH subarachnoid extension, n (%) | 27 (10.6) | 14 (9.6) | 13 (11.9) | 0.693 |

**Supplementary Results:**

***Supplementary table 1. Included and excluded ICH survivors profiles***

***.***IQR = interquartile range; IS = ischemic stroke; ICH = intracerebral haemorrhage; mRS = modified Rankin Scale; NIHSS = National Institutes of Health stroke scale; ^a^Expressed in mL.

***Type and change of headache during follow-up of patients with previous history of headache:***

Of the 19 patients that had both previous history of headache and headache at 1-year after ICH, 7 had history of migraine, 6 had history of tension headache, and 6 had history of headache of unspecified type. At 1-year follow-up of the 7 patients with history of migraine, 4 (57%) had migraine, 1 (14%) had tension headache, and 2 (29%) had headache of unspecified type. At 1-year, 3 of the migraineurs (43%) reported diminished frequency/intensity of migraines, 2 (29%) reported disappearance of migraines, 1 (14%) reported intensification of migraines, and 1 (14%) had missing data on change in headache intensity. At 1-year follow-up of the 6 patients with history of tension headache, 6 (100%) had tension headache. At 1-year, 4 patients (67%) reported intensification of tension headaches, 1 (17%) reported diminished frequency/intensity of tension headaches, and 1 (17%) had missing data on change in headache intensity.

***Supplementary figure 1. Depressive symptoms severity at 1-year follow-up for patients with and without post-ICH headache.***


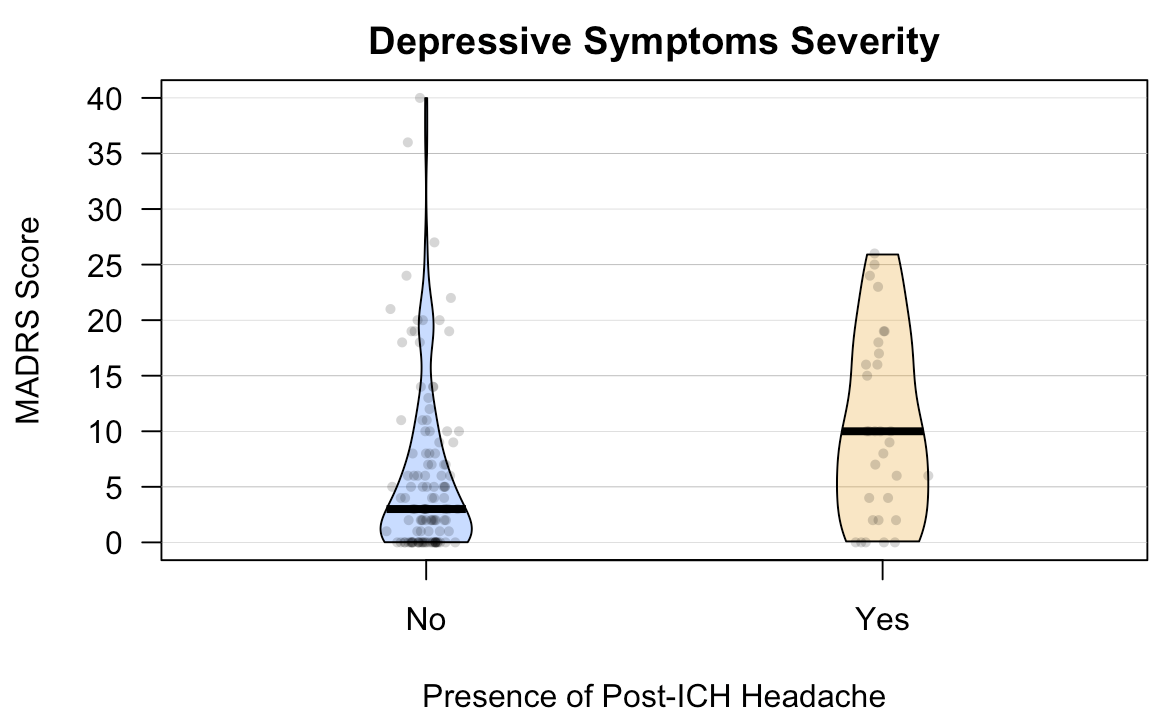


Depressive symptoms severity was assessed using the Montgomery-Åsberg Depression Rating Scale (0-60). The pirate plot was created using the YaRrr package on R. The points represent raw data; the bold bars denote group medians; and the beans display smoothed distributions of data density.

***Supplementary figure 2. Anxiety symptoms severity at 1-year follow-up for patients with and without post-ICH headache.***

**
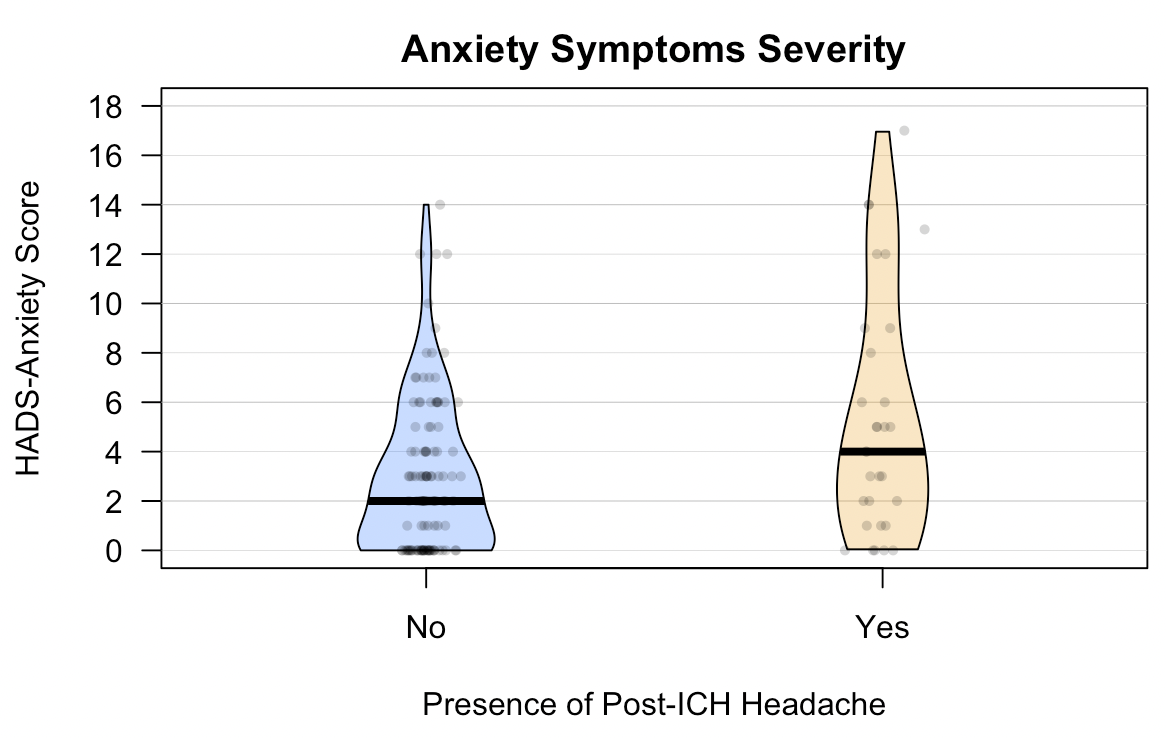
**

Anxiety symptoms severity was measured using the Anxiety subscale of the Hospital Anxiety and Depression Scale (0-21). The pirate plot was created using the YaRrr package on R. The points represent raw data; the bold bars denote group medians; and the beans display smoothed distributions of data density.

***Supplementary table 2. Additional patient characteristics and headache at 1-year follow-up.***

| **Baseline clinical and radiological features** | **Total**  **(n = 146)** | **Without headache**  **(n = 115)** | **With headache**  **(n = 31)** | **p-value** |
| --- | --- | --- | --- | --- |
| Probable CAA, n (%) | 21 (14.4) | 17 (14.8) | 4 (12.9) | 1 |
| Surgical treatment of ICH, n (%) | 7 (4.8) | 4 (3.5) | 3 (9.7) | 0.165^*^ |
| IVH extension, n (%) | 40 (27.4) | 31 (27.0) | 9 (29.0) | 0.998^*^ |
| **1-year follow-up characteristics** | | | | |
| Cognitive impairment, n (%) | 34 (24.1) | 27 (24.3) | 7 (23.3) | 1 |

Probable CAA was defined using the Boston Criteria version 2.0.^4^ Cognitive impairment was defined as a score ≤ 23 on the Mini-Mental State Examination at 1-year follow-up.^5^

^*^Fisher’s Exact Test performed.

CAA = cerebral amyloid angiopathy; IVH = intraventricular haemorrhage

***Supplementary table 3. Multivariable logistic regression model comprising baseline factors and previous conditions.***

| ***Headache at 1-year follow-up: baseline predictors*** | | |
| --- | --- | --- |
| ***Independent variables*** | ***Odds ratio (95% CI)*** | ***p-value*** |
| Age at baseline | 0.98 (0.95-1.02) | 0.302 |
| Previous history of headache | 4.60 (1.74-12.1) | 0.002 |
| Headache at ICH onset | 2.75 (1.02-7.42) | 0.045 |

Presence of headache at 1-year follow-up is the dependent variable.

**Supplementary References:**

1. Cordonnier C, Rutgers MP, Dumont F, Pasquini M, Lejeune JP, Garrigue D, Béjot Y, Leclerc X, Giroud M, Leys D, et al. Intra-cerebral haemorrhages: are there any differences in baseline characteristics and intra-hospital mortality between hospitaland population-based registries? *J Neurol* [Internet]. 2009 [cited 2022 Aug 30];256:198–202. Available from: https://pubmed.ncbi.nlm.nih.gov/19271106/

2. Moulin S, Labreuche J, Bombois S, Rossi C, Boulouis G, Hénon H, Duhamel A, Leys D, Cordonnier C. Dementia risk after spontaneous intracerebral haemorrhage: A prospective cohort study. *Lancet Neurol*. 2016;15:820–829.

3. Kothari RU, Brott T, Broderick JP, Barsan WG, Sauerbeck LR, Zuccarello M, Khoury J. The ABCs of measuring intracerebral hemorrhage volumes. *Stroke* [Internet]. 1996 [cited 2022 Aug 31];27:1304–1305. Available from: https://pubmed.ncbi.nlm.nih.gov/8711791/
